# Tribological Performance of Nylon Composites with Nanoadditives for Self-Lubrication Purposes

**DOI:** 10.3390/polym12102253

**Published:** 2020-09-30

**Authors:** Isabel Clavería, Sofía Gimeno, Ignacio Miguel, Gemma Mendoza, Aleida Lostalé, Ángel Fernández, Pere Castell, Daniel Elduque

**Affiliations:** 1 Mechanical Department, EINA, University of Zaragoza Maria de Luna, 50018 Zaragoza, Spain; 547686@unizar.es (A.L.); afernan@unizar.es (Á.F.); delduque@unizar.es (D.E.); 2 FERSA BEARINGS S.A., Bari, 50197 Zaragoza, Spain; sofia.gimeno@fersa.com (S.G.); ignacio.miguel@fersa.com (I.M.); 3 Fundación Tekniker, Iñaki Goenaga, 20600 Eibar, Spain; gemma.mendoza@tekniker.es; 4 Fundación AITIIP, Calle Romero, 50720 Zaragoza, Spain; pere.castell@aitiip.com

**Keywords:** ZrO_2_, graphene, MoS_2_, friction, wear, polyamide

## Abstract

A systematic study comparing the wear behaviour of composites with nylon matrix (PA66, PA46, PA12) and different nanoadditives and reinforcing additives (graphite, graphene, MoS_2_ and ZrO_2_) has been carried out in order to achieve a proper self-lubricant material for bearing cages. The wear characterisation was done using pin-on-disc tests, SEM and EDX analysis. The results show that better outcomes are obtained for composites based on PA12. The addition of ZrO_2_ offers negative values of wear due to the metallic particle transference from the counterface to the polymeric pin.

## 1. Introduction

One of the challenges that has arisen during recent years in the industrial field is to make industrial processes and component performance more sustainable. In this sense, one of the most used industrial components is bearings, so improving their energy efficiency may be key to reduce fuel consumption and pollutant emissions. As an example, recent analyses [1] show that for every litre of fuel used in an average vehicle, 5% is consumed in mechanical losses and 1% of the total is lost due to bearing operation. Since a significant part of the energy required for industrial component performance is lost in form of heat due to friction [2], new strategies are required to develop low-friction components. Traditionally, these strategies have been focused on modifying surfaces through different treatments to improve surface hardness [3,4], or to develop hard coatings deposited by physical [5] or chemical vapor deposition (PVD and CVD) [6], and the development of new lubricants [7]. However, some friction and wear phenomena are characterised by different scale processes (ones at atomic or molecular scales and others at a scale of roughness of approximately 1–20 μm [8]) that operate simultaneously. This fact leads to difficulties in studying microscopic phenomena resulting in macroscopic wear and friction and becomes one the biggest challenges of current tribology research. Furthermore, problems related to coating detachment can arise because adherence properties strongly depend on the morphology and composition of the coating layer [5], and also on the interacting forces between the coating layers and the substrate surface. Therefore, the development of new self-lubricant materials without coating requirements seems to be an excellent solution to achieve low-friction properties, minimising maintenance costs due to the presence of additional lubricants.

As stated previously, bearings, and particularly bearing cages, are one of the mechanical applications where low-friction polymers can play an essential role in order to achieve a high performance under high speed and temperature conditions by means of reducing the wear between the cage and rolling elements, as investigated by Fersa Bearings S.A. [5,6,7,8,9] and other authors [10,11].

Polymers have traditionally been investigated for industrial purposes, especially in mechanical components subjected to high speed, because of their light weight and, thus, their low inertial contribution.

Some polymers have also been investigated in relation to their excellent tribological properties, such us nylons (PA) [12,13] and polytetrafluoroethylene (PTFE) [14,15]. However, due to the poor mechanical properties of PTFE, it has usually been used as a self-lubricant additive into other polymeric matrices rather than as a polymeric matrix itself [16,17,18,19]. Nylons stand out thanks to their ability to form a proper transfer film when sliding against metallic counterparts [20,21]. PA66 is one of the most commonly used nylons for mechanical purposes [22,23], as well as other relatively new engineering nylons, such as PA12 and PA46 [24]. In the case of PA46, it is expected to show superior performances to other nylons used in engineering parts and components, such as gears, because of its higher modulus and strength as a result of being highly crystalline.

Nevertheless, the engineering performance of nylons can be improved by means of developing new composites, combining the optimum processability of polymers and the excellent wear and mechanical properties of some solid lubricant nanoadditives. Nanostructures derived from carbon allotropes such as graphite and graphene have been widely proven to provide good properties for mechanical and tribological purposes in several polymeric matrices, such as PA [22,23,24,25,26,27], PTFE [28], PEEK [29] or PES [30].

ZrO_2_ can be used as a nanocomponent, usually added to metal and cermet matrices to improve its mechanical and wear behaviour [31,32], or it can also be used as a direct coating for metallic bare substrates [33]. However, few examples are found in literature about the wear performance of polymeric composites containing ZrO_2_ [9], and some of the studies refer only to polymeric composites including other components apart from ZrO_2_. The results reported by these studies are variable regarding the performance of ZrO_2_ in the wear behaviour of the composite, as described in [9,34,35]. Despite these results, few investigations have focused on the improvement of the tribological properties of polymers doped with ZrO_2_ and, consequently, this paper aimed to increase the knowledge of this nanocomponent and its behaviour working as a filler in different polyamides.

Due to its lamellar shape made of individual atomically thin planes that can easily slide against each other [36], molybdenum disulphide (MoS_2_) has been successfully used as a solid lubricant. MoS_2_ can work, alone or with other additives, in different environments, such as: polymeric composites based on PEEK [37,38], ABS [39], PI [40] and PA [19,41,42,43,44]; liquid lubricants [45,46,47] or coatings [48,49,50], showing promising results in terms of improving friction coefficient and wear rate.

In many of the studies found in the literature, the polymer composites are formed by a combination of several nanoadditives mixed in the same matrix, such as PA12-graphene-paraffine oil in [26]; PEEK-ZrO_2_-Ni in [34]; PEEK-graphene-WS_2_-CNT in [29]; PI-MoS_2_-graphene in [40]; PA6-TiO_2_-MoS_2_ in [41]; PPSK-graphite-PFTE in [51].

With this background, a systematic study comparing potential solid lubricants for the same polymeric matrix under similar conditions is missing. It seems to be suitable to carry out a study that provides comparable results about the tribological behaviour of some polyamides doped with some of the nanoadditives that have exhibited some wear improvement.

The aim of this research is to provide comparative results about the wear behaviour of different solid lubricants such as graphite, graphene, MoS_2_ and ZrO_2_ with the same polymeric matrix (PA66, PA46 and PA12) under similar test conditions.

## 2. Experimental

### 2.1. Materials

Polymer nano-reinforced composites based on PA66 from Bada Hispanaplast S.A. (PA66 BADAMID A70S, Huesca, Spain) [52], PA46 from DSM Engineering Plastics (STANYL^®^ TW341, Heerlen, Netherland) [53] and PA12 from Bada Hispanaplast S.A. (PA12 BADAMID PA12H) [54] were analysed.

The different polyamides were filled with different additives in order to obtain enhanced tribological properties. In particular, four different fillers were used: ZrO_2_, MoS_2_, graphene and graphite. The graphene used was Avangraphene Plat 40 (Avanzare, Navarrete, Spain) which is a two-dimensional carbon atom structure obtained via the mechanical exfoliation of graphite, with an oxygen content (X-ray photoelectron spectroscopy) of <0.5%, a hydrogen content of <1%, a nitrogen content of <1%, an average thickness of 10 nm, a width (laser diffraction) of 40 μm, a surface area (Brunauer–Emmett–Teller) of 282 m^2^/g and an average number of layers of < 30. It is specially designed to be mixed with polymeric materials, and a low dosage is required to reach the percolation level. General purpose ZrO_2_ supplied by Torrecid S.A. (Castellón, Spain) [55], with a size of D50 1.20 ± 0.20 μm (spherical) and with a tensile strength of 450 MPa has also been used. The laminar MoS_2_ used was M15 with an average size of D50 = 1.5 μm and a thickness of 10 nm from Brugarolas S.A (Barcelona, Spain) [56]. Two-dimensional graphite, with a similar structure to MoS_2_, was provided by GrapheneTech S.L. (Zaragoza, Spain) [57].

### 2.2. Specimen Preparation

In order to characterise the different composites, the tribological tests were performed using a pin-on-disc configuration. Overall, twelve different nano-reinforced composite materials were prepared, as shown in Table 1. The samples were prepared via extrusion compounding in a co-rotating twin-screw extruder (Coperion ZS26K, Barcelona, Spain) equipped with two gravimetric feeders (Brabender Technologies, Duisburg, Germany). All the samples were prepared using the same experimental conditions: temperature profile (270 °C to 300 °C at the die), screw speed between 100 and 175 rpm, torque between 60 and 80 Nm and pressure between 80 and 85 bar. The screw profile was designed to achieve a high to medium shear profile that was optimised in previous studies [58,59]. A screw diameter of 25 mm and a length/diameter ratio of 40 were used in the different test procedures.

The optimum level of additive dispersion into the polymeric matrix was determined using an ultrasonic device specially designed for this purpose, fully described in previous studies [58,59]. These studies varied the concentration of nanoadditives and the extrusion conditions in PA6 matrices. Once the materials were processed, mechanical, thermal and morphological studies determined the optimal concentration of additives, which resulted in the best combined properties. The dispersion of the additives determined by Scanning Electron Microscope (SEM) and Transmission electron microscope (TEM) was key to selecting the optimal conditions. From these studies, the authors determined and selected the optimal loading that has been used in the present study.

On the one hand, the content of additives was determined by weight in all the formulations prepared. On the other hand, the quantity of additives was set at 5% since this percentage shows promising results in the literature [9].

Pins of 8 × 8 × 4 mm^3^ from the resulting composite materials were injected using an electric injection machine (JSW 85 EL II, Tokyo, Japan) with a clamping force of 85 tons, a maximum injection pressure of 1960 bar, a maximum injection volume of 97 cm^3^ and a screw diameter of 32 mm. The process conditions were as follows: an injection temperature profile of 240/260/280 °C, a mould temperature of 70 °C, an injection time of 1 s, a packing pressure of 50 MPa for 5 s and a cooling time of 7 s.

As the purpose of the study was to obtain a self-lubricating material for bearing cages, the disc specimens (dimensions Ø24 × 7.9 mm) were made of an AISI 52,100 100Cr6, the material used in rolling bearings, with a hardness of HRC 59–63 and a roughness of R_a_ < 0.15 μm according to the Fersa Bearings S.A. specification.

### 2.3. Pin on Disc Tests

**Figure 1:**
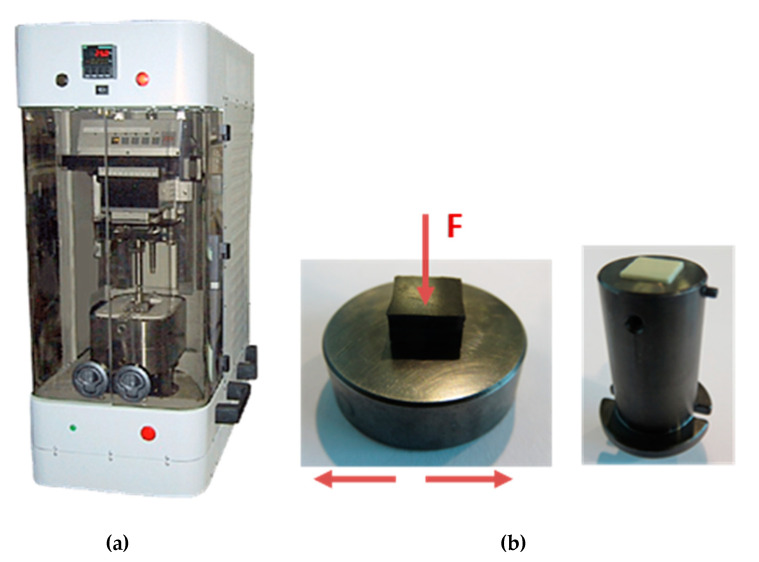
(**a**) CETR-UMT tribometer and (**b**) pin-on-disc testing configuration.

The tests were performed on a CETR-UMT tribometer using a polymeric pin oscillating at a constant frequency (5 Hz), a stroke amplitude (4.25 mm) and under a defined load (384 N), against a stationary steel test disc under dry conditions for 1800 s. The sliding speed was 42.5 mm/s. A scheme of the testing configuration is shown in Figure 1.

The following results were recorded: the coefficient of friction (CoF) evolution over time, obtained using CETR-UMT tribometer software (Viewer of UMT) which processes measured values, and the wear determined by the samples’ mass loss, weighed using an electric balance in order to calculate the specific wear rate via the following equation:

(1) $$w_{s}=\frac{∆m}{\rho F_{N}L}\left(\frac{mm^{3}}{Nm}\right)$$

where *F_N_* is the normal force applied during the test, *ρ* is the material density, *L* = 76.5 m is the sliding distance according to the sliding speed and test duration and ∆*m* is the mass loss obtained experimentally. Density values (*ρ*) are calculated from the equation:

*ρ* = (%_matrix_ × *ρ*_matrix)_ + (%_additive_ × *ρ*_additive_) (2)

where *ρ* is the density for the sample material, %_additive_ is the mass percentage of additive added to the sample composite, %_matrix_ is the mass percentage of polymer material, *ρ*_matrix_ is the density of the polymer and *ρ*_additive_ is the density of the additive.

### 2.4. SEM and EDS Analysis

The microstructure of some of the samples was studied using High-Resolution FE-SEM Ultra Plus Zeiss equipment (Jena, Germany), a scanning electron microscope with field emission gun. The equipment has a complete detection system (EsB^®^ detector with filtering grid (0–1500 V), a high efficiency in-lens SE detector, a chamber-mounted Everhart–Thornley detector, an Integrated AsB^®^ detector EBS, SE). The EDS Oxford INCA Energy 350 system was used for microanalysis. Energy dispersive X-ray spectroscopy (EDS) allowed for elemental and chemical analysis of the samples.

## 3. Results and Discussion

### 3.1. Coefficient of Friction and Wear

**Figure 2:**
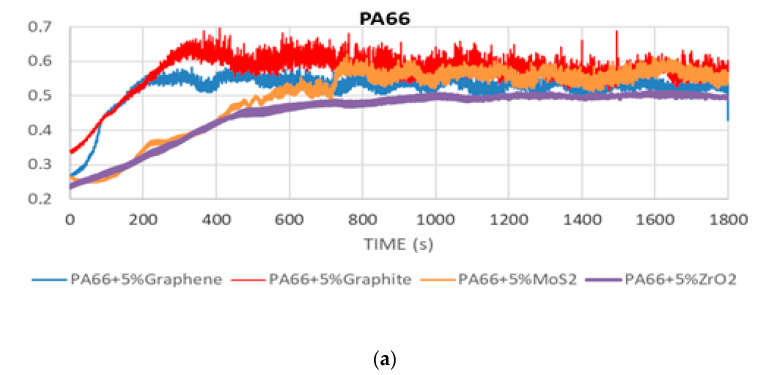

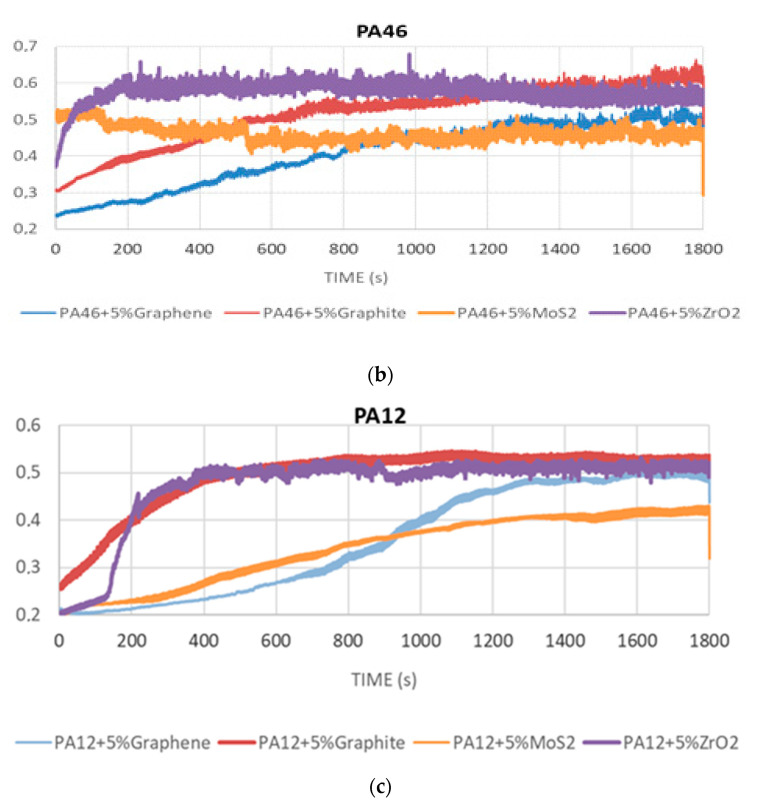
CoF evolution for composites based on (**a**) PA66, (**b**) PA46, and (**c**) PA12.

Figure 2 shows the evolution of the CoF during the wear test for the hybrid composite materials described in Table 1.

The results exhibit an increasing trend of the CoF during the running-in stage for all composites. Then, once the transfer film is created and remains intact, the curves reach a steady stage, exhibiting constant values [60,61].

Lower values of CoF are achieved with ZrO_2_, graphene and MoS_2_ in the case of the PA66 (Figure 2a) composites, obtaining values of 0.49, 0.52 and 0.53, respectively. PA66 samples doped with graphite have higher CoF values (μ = 0.59). Regarding PA46 composites, lower values of CoF are achieved for samples doped with MoS_2_ (μ = 0.45). PA46 samples (Figure 2b) doped with graphene show a CoF value of 0.5, and those doped with graphite and ZrO_2_ have higher friction coefficients (μ = 0.57 and 0.58, respectively). In the case of PA12-based composites (Figure 2c), almost all the samples show lower CoF values than the rest of the composites for each additive. The low values of CoF achieved for samples doped with MoS_2_ (μ = 0.41) are remarkable.

Table 2 summarises the average values of the CoF, which were calculated by averaging the experimental values from the steady-state stage in Figure 2, as well as the specific wear rate *w*_s_, calculated using Equation (1).

According to Table 2, mass loss and, thus, its wear rate are reduced for the sample composites based on PA46 and PA12, achieving values under 0.003 for the wear rate in most of the samples. PA66 composites exhibit a worse behaviour with mass loss values over 0.24; 0.35 in the case of graphene, 0.25 in the case of graphite and 0.24 in the case of MoS_2_. The role of ZrO_2_ nanoparticles in all the composites is remarkable, leading to negative values of mass loss in the polymeric pin (zero mass loss in the case of PA66, −0.06mg in the case of PA46 and −0.09 mg in the case of PA12). That is the reason why *w*_s_ is not calculated for composites with ZrO2 nanoparticles in Table 2. This fact indicates that polymeric pins increase their mass instead of reducing it, and it will be further analysed. Again, the best results are achieved by the addition of 5% MoS_2_ to PA12, reducing the wear rate up to 0.01.

It can be stated that composites with PA12 and PA46 matrices exhibit a better tribological behaviour than the rest of the composites, with lower CoF values and lower wear rates. Since polymers are viscoelastic materials, the material softens because of the relaxation of molecular chains as temperature increases during the friction process in the contact area. As a consequence, Young’s modulus decreases, leading to a higher plastic deformation and a higher real contact area, making the adhesive wear mechanism dominant, reducing the wear resistance and increasing the CoF until a transfer layer is constituted [62]. On the other hand, the shear strength is also reduced by increasing the frictional heat, which contributes to the increment of the CoF. In the case of the PA46 matrix, its molecular structure is characterised by a higher number of functional groups in the polymeric chain, which form strong hydrogen bonds, as well as the highest amide group density of all commercial polyamides, resulting in a higher melting temperature [63]. Since the wear mechanism for polymers is mainly adhesive, and the wear is caused by frictional heat between the two contact surfaces, PA46 is capable of reducing damage thanks to the higher melting temperature and a less adhesive wear, due to a lower plastic deformation and a lower increase in contact area. On the other hand, both matrices, PA46 and PA12, have a higher crystallinity, near 70%, which keeps Young’s modulus very high (3300 MPa for P46 [53] and 1700 MPa for PA12 [54]), even up to temperatures near their melting point, avoiding higher levels of plastic deformation [64] and thus reducing the wear. In the case of the PA66 matrix, a more severe wear is achieved corresponding to the generation of higher temperatures on the sample surfaces due to the increased frictional heating.

It seems that the transition point is associated with the softening temperature of the sample material. Provided that the surface temperatures with PA12 and PA46 matrices are below this level, mild wear occurs with very low rates of material loss for PA46 and PA12 composites. Above this level, a more severe wear is found for PA66 composites.

### 3.2. Influence of Nanoadditives

It has been demonstrated that incorporating nanoadditive contents into the polymer matrix modifies the wear mechanism [9]. This means that the particles incorporated in the polymer effectively act to restrain the direct contact between the polymer and the hard steel counterpart, owing to their good solid lubricating and easy shearing action [51]. Subsequently, the friction and the wear are seriously decreased regarding non-doped materials. The presence of nanoadditives plays two main roles in order to improve wear properties. Firstly, the thermal conductivity of some nanoadditives is much higher than the thermal conductivity of the polymeric matrix (0.1–0.4 W/mK for nylons, >3000 W/mK for graphene [65], 300 W/mK for graphite [66], 85 W/mK for MoS_2_ [67] and ≈ 2 W/mK for ZrO_2_ [68]). This fact and the large surface area of the nanofillers show that the heat between the contact surfaces can be easily dissipated during the wear process. Therefore, as a consequence of a weaker softening of the polymeric matrix, severe adhesive wear is reduced.

Secondly, the presence of nanoadditives in polymeric matrices prevents crack propagation generated during the sliding process, thanks to the efficient stress transfer from the polymer matrix to the nanoadditives, leading to a mild wear mechanism [69]. On the other hand, according to [44], a material can be worn in a chemical and/or mechanical way, and the heat generated during the wear process can contribute to it. In the case of MoS_2_ particles, Mo atoms can become oxidised to MoO_3_, and S atoms can become oxidised and react with the Fe substrate, turning into FeS. This enhances adhesion between the counterpart and the transfer film and prevents its loss, improving the wear properties of the MoS_2_ composite [70].

These observations indicate that the proposed fillers, in small quantities, were helpful for a wear reduction, as has been supported in the literature [9].

### 3.3. Characterisation of the Worn Surface

**Figure 3:**
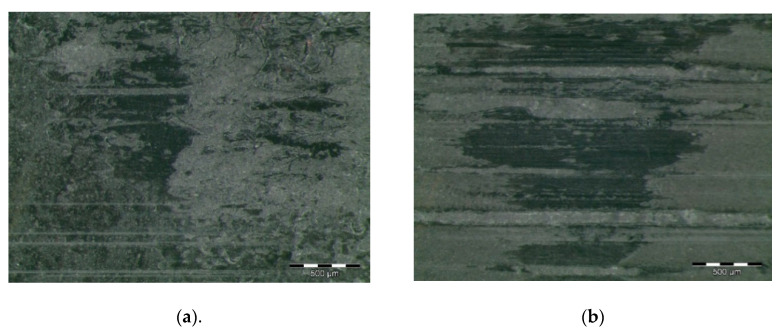

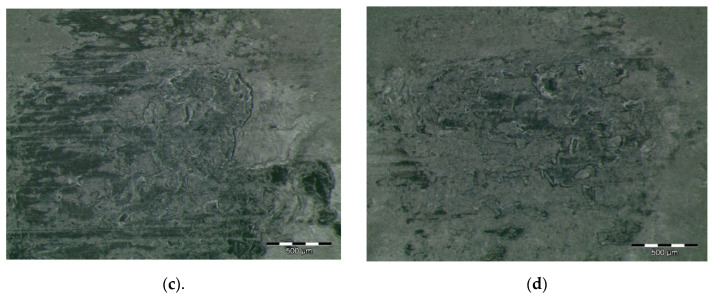
Worn surfaces of PA66 composites: (**a**) PA66 + 5% graphene; (**b**) PA66 + 5% graphite; (**c**) PA66 + 5% MoS2; (**d**) PA66 + 5% ZrO_2_.

Figure 3 shows worn surfaces of pins based on a PA66 matrix. A top layer disintegration is observed in Figure 3a,b for samples doped with graphene and graphite. Removed material appears in the form of blocks, indicating an adhesive wear mechanism. Figure 3a shows a smoother worn surface, which reveals that the graphene particles strongly protect the top of the composite material surface under dry sliding test conditions. There is not any significant crack formation, broken particles or wedge formation, which means good bonding between the graphene filler and the matrix material. Graphene particles become integrated in the PA66 composites and, consequently, the worn surface is protected from the wear and friction. This may be due to the excellent self-lubricity of graphene that reduced the friction and the wear of the polymer composites [9]. Figure 3b shows the back transfer of the material in a patchy form, indicating an adhesive wear, as well as some mild abrasive wear with shallow grinding marks. Nevertheless, the severity of the wear mechanism is reduced due to the presence of graphite. The bonding strength of graphite is appreciable on the surface of the composites, indicating the migration action and the layer formation of graphite with a polymeric matrix, although weak wear track formation is also visible [71,72].

Figure 3c shows a relatively rough worn surface of a sample doped with MoS_2_, with some matrix damage and weak wear tracks, which also confirm the adhesive wear mechanism. In the same way, slight plastic deformation is observed in Figure 3d for PA66Z. Nevertheless, ZrO_2_ particles could interrupt the material deformation during the wearing process and hinder the propagation of potential cracks, and thus reduce the wear rate and the coefficient of friction. Additionally, the formed smooth worn surface can facilitate the sliding movement by reducing the adhesion and abrasive wear, hence protecting the polymeric matrix.

In the case of samples based on a PA12 matrix, the distribution of graphene nanoplatelets results in a high surface mechanical strength. The high surface mechanical strength helps in preventing deep wear grooves during the sliding action that appear in non-doped PA12 [73].

**Figure 4:**
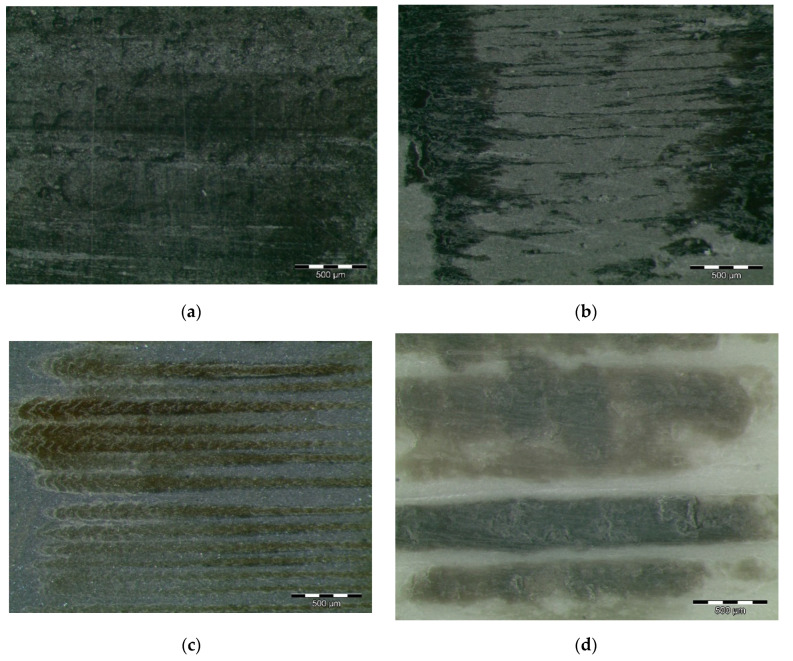
Worn surfaces of PA12 composites: (**a**) PA12 + 5% graphene; (**b**) PA12 + 5% graphite; (**c**) PA12 + 5% MoS_2_; (**d**) PA12 + 5% ZrO_2_.

Besides the surface properties, the transfer films formed during sliding also play a significant role in controlling the wear behaviour of the materials [9]. During the sliding process, these graphene nanoplatelets were easily released from the PA12 nanocomposites and transferred between the PA12 nanocomposites contact zone and the abrasive counterface. Thus, graphene nanoplatelets could work as a solid lubricant material between the two contacted surfaces and prevent direct contact between them, thereby reducing the coefficient of friction and increasing the wear resistance. This resulted in a mild wear mechanism with fewer marks on a PA12GF pin surface, as shown in Figure 4a. Figure 4b shows that PA12GT abraded surfaces are less smooth than samples doped with graphene, as shown by the initiation of cracking on the worn surface. This phenomenon can be attributed to the presence of severe plastic deformation during the friction and wear tests. Accordingly, the wear mechanism can be defined as adhesive and slightly abrasive. Figure 4c shows some spiky marks indicating the presence of a tribofilm formed with the MoS_2_. In the sample of PA12Z shown in Figure 4d, some crater grooves appear, indicating that some ZrO_2_ particles detached and caused damage in the PA matrix. The dark colour can be attributed to steel material from the counterface.

**Figure 5:**
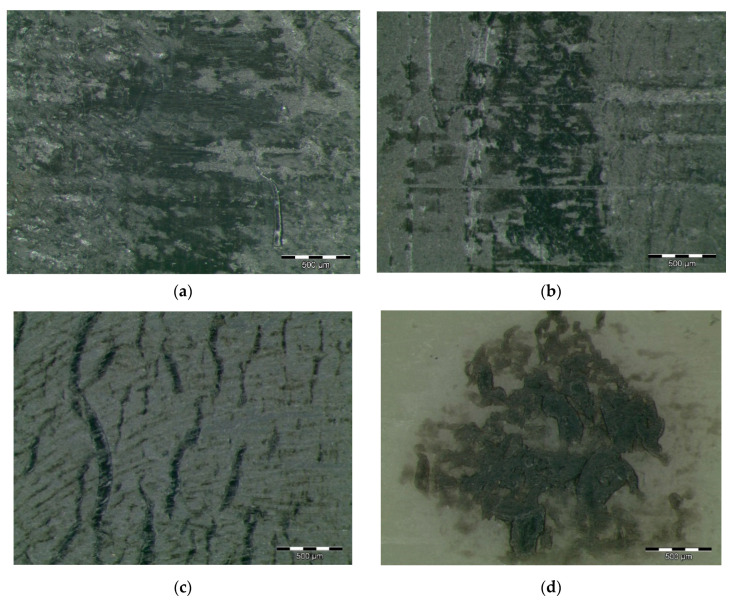
Worn surfaces of PA46 composites: (**a**) PA46 + 5% graphene; (**b**) PA46 + 5% graphite; (**c**) PA46 + 5% MoS_2_; (**d**) PA46 + 5% ZrO_2_.

Similar to samples of PA66 doped with graphene and graphite, an adhesive wear mechanism is activated for PA46 pin surfaces, as shown in Figure 5a,b, exhibiting removed material from the surface in the form of blocks. Regarding samples doped with MoS_2_, in Figure 5c, transverse cracks, spaced unevenly, are seen across the wear surfaces of the PA46 + MoS_2_ samples as well as the formation of polished surfaces and areas of melting, indicating a severe adhesive wear. Tensile stresses produced in the contact area and the high surface temperatures created play a significant role in the crack formation. These worn surfaces reflect some typical fatigue deformations with deep cracks and compact furrows. Figure 5d shows local plastic deformation in the contact area for samples of PA46 doped with ZrO_2_, confirming an adhesive wear mechanism.

Since PA46 has better mechanical properties than the rest of the matrix, which are required for global bearing cage design, we selected PA46 composites to perform a deeper analysis to better understand the influence of nanoadditives and transfer film generation after a pin-on-disc test. PA46 samples without nanoadditives were tested in the same conditions as the rest of the samples, obtaining a value of CoF of 0.62, which is coherent with previous literature [74,75]. The average coefficient of friction values reported for PA46 samples with nanoadditives are much lower than these values, demonstrating the positive effect of nanoadditives in reducing the CoF value significantly.

Given the results presented above, as previously stated, a deeper analysis of PA46 composites was performed in order to better understand transfer film generation. SEM images were obtained and EDS analysis was carried out on discs to check sample composition.

**Figure 6:**
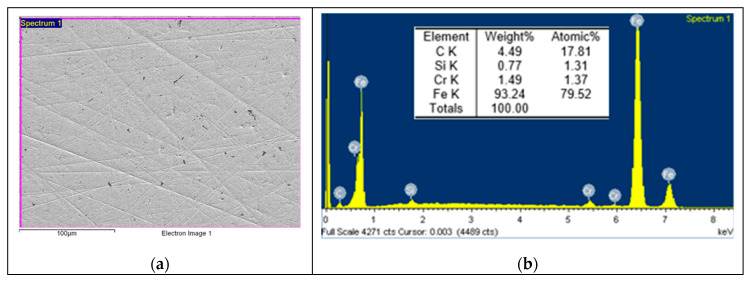
(**a**) SEM image and (**b**) EDS spectrum for non-tested discs.

**Figure 7:**
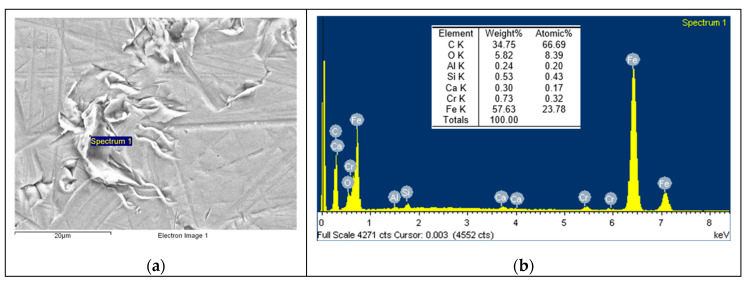
(**a**) SEM image and (**b**) EDS spectrum for discs tested against the non-doped PA46 sample.

**Figure 8:**
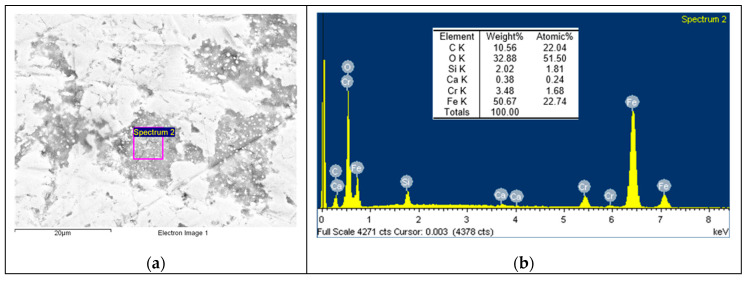
(**a**) SEM image and (**b**) EDS spectrum for discs tested against the PA46GT sample.

**Figure 9:**
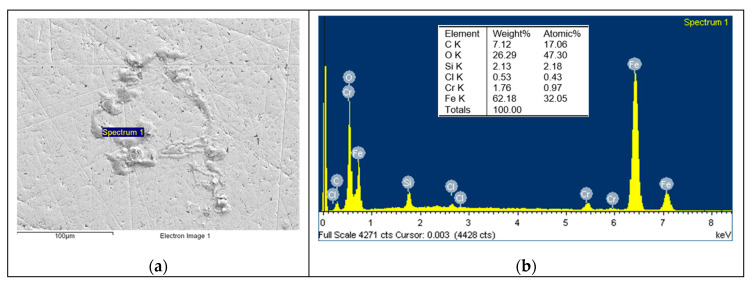
(**a**) SEM image and (**b**) EDS spectrum for discs tested against the PA46GN sample.

**Figure 10:**
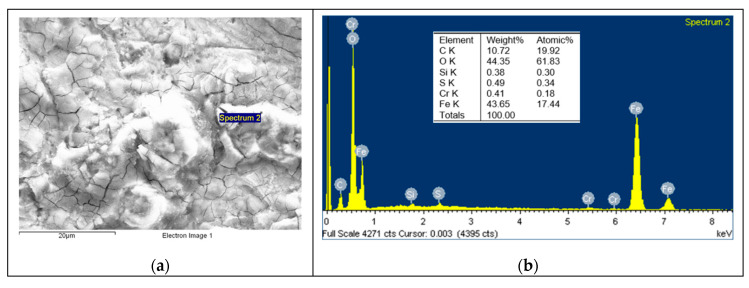
(**a**) SEM image and (**b**) EDS spectrum for discs tested against the PA46M sample.

**Figure 11:**
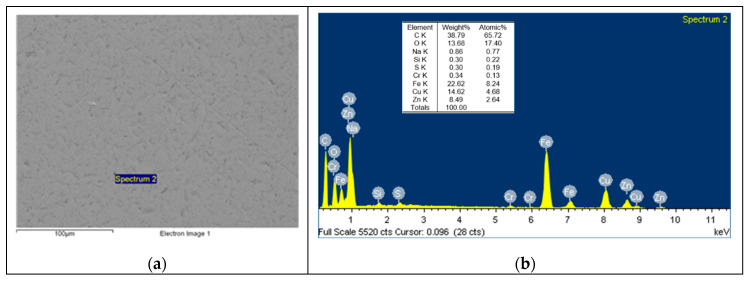
(**a**) SEM image and (**b**) EDS spectrum for discs tested against the PA46Z sample.

Based on the EDS spectrum results, summarised in Table 3, it can be concluded that, for non-doped PA46, transfer film is formed after the pin-on-disc test since the carbon quantity increases from 4.49, in Figure 6 for non tested disc, to 34.75% and oxygen appears with an amount of 5.82% in Figure 7. The same phenomena can be observed for PA46GT in Figure 8, where carbon weight changes from 4.49% to 10.56% and oxygen emerges with 32.88%. In PA46GN (Figure 9), tribofilm is also formed since the carbon quantity rises up to 7.12% and oxygen surfaces with a weight of 26.29%. When analysing PA46M in Figure 10, transfer film formation can be confirmed not only by a carbon quantity increase (from 4.49% to 10.72%) and oxygen presence (44.35%), but sulphur also arises with a content of 0.49%. Finally, concerning PA46Z samples in Figure 11, the rise of carbon weight grows from 4.49% to 38.79% and the emergence of oxygen by 13.68%, which leads to the conclusion of tribofilm formation.

### 3.4. Analysis of Wear in ZrO_2_ Composites

**Figure 12:**
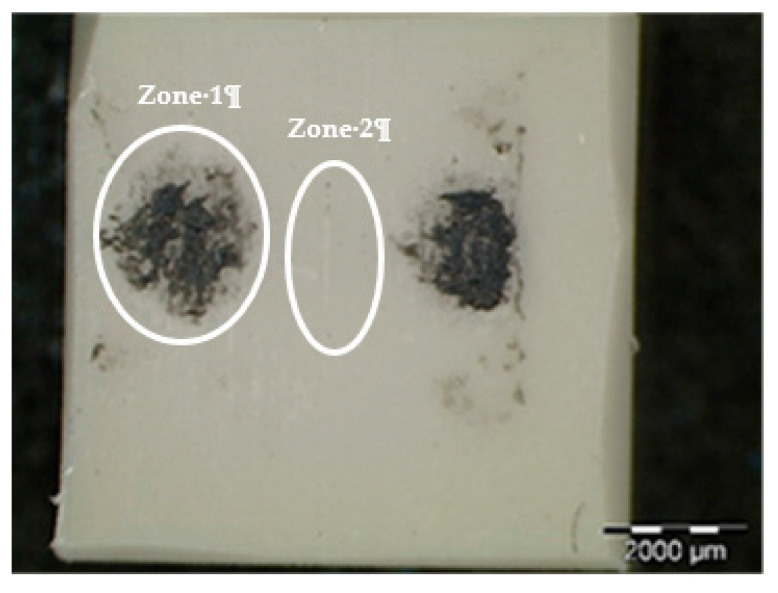
Surface of PA46Z after testing.

The negative wear values obtained in the tests and shown in Table 2 could be attributed to the transfer of metallic particles of the 100Cr6 disc to the polymeric pin. The dark colour in Figure 5d also could indicate the presence of steel material from the counterface. Since PA46Z samples show the highest negative mass loss, they were subjected to a further analysis of their surface and composition by means of SEM and EDS. Figure 12 shows the surface aspect of a pin on which two different zones, representative of the main wear mechanism produced in the pin surface, were analysed (zone 1 and zone 2). These two different zones are selected for further analysis because the contact of the pin against the counterface is not exactly the same at zone 1 and zone 2, as can be appreciated in Figure 12. Since the sample pin surfaces are not entirely flat and the pin could not be properly aligned during the test, zone 1 and 2 do not contact in the same way against the counterface, and they should both be studied.

**Figure 13:**
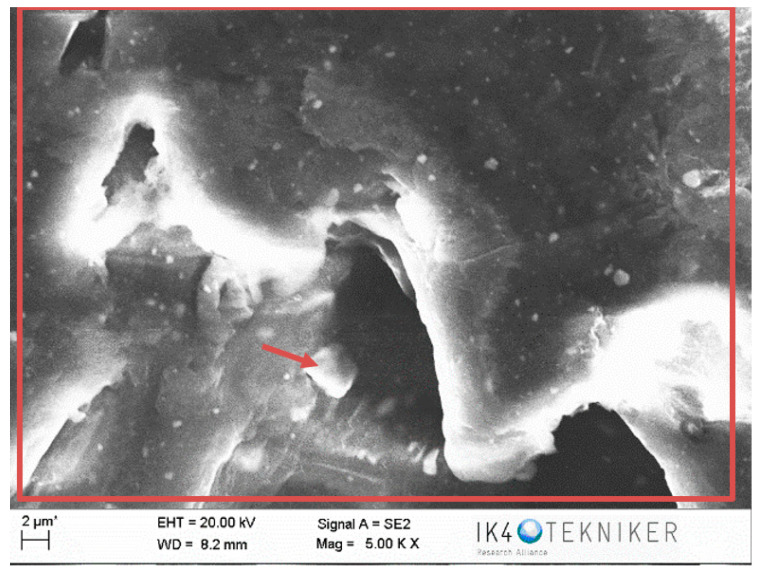
SEM image for zone 1 in the PA46Z sample.

**Figure 14:**
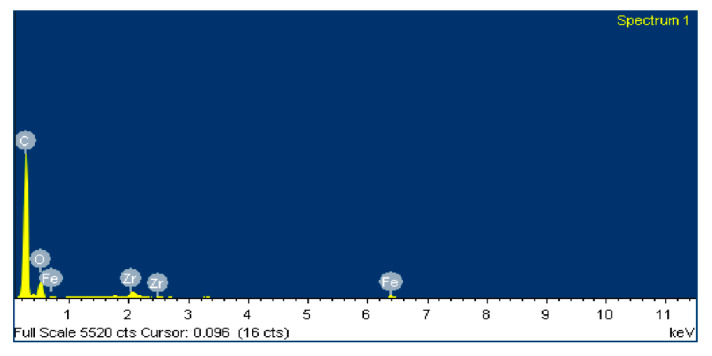
EDS spectrum for zone 1 in the PA46Z sample.

**Figure 15:**
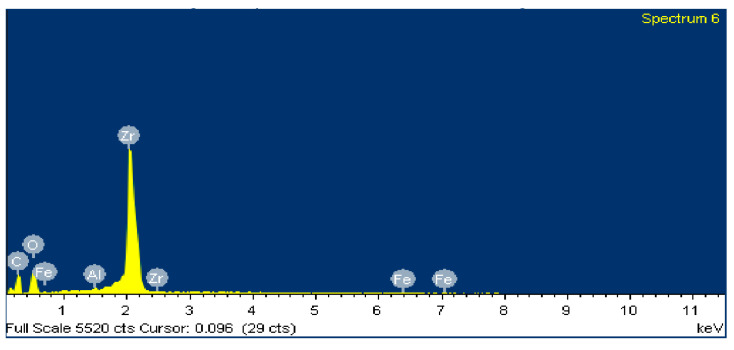
EDS spectrum for zone 1 in a local area marked with an arrow in Figure 13, where ZrO_2_ is located.

**Figure 16:**
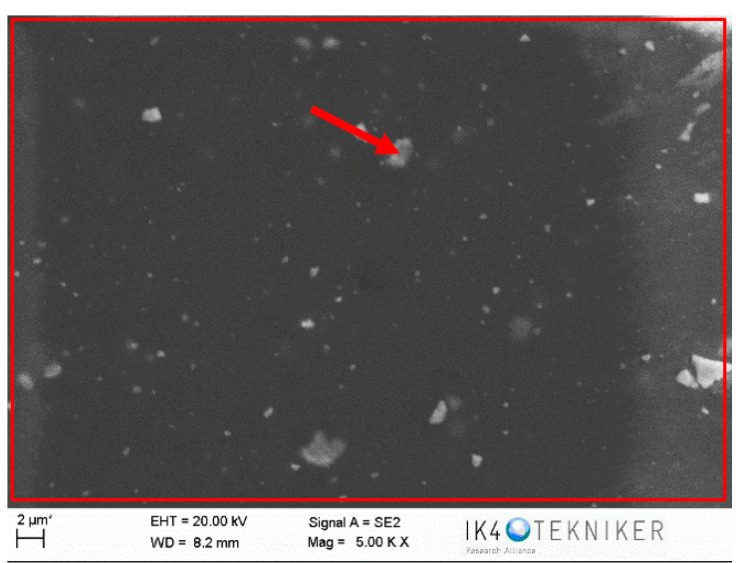
SEM image for zone 2 in the PA46Z sample.

**Figure 17:**
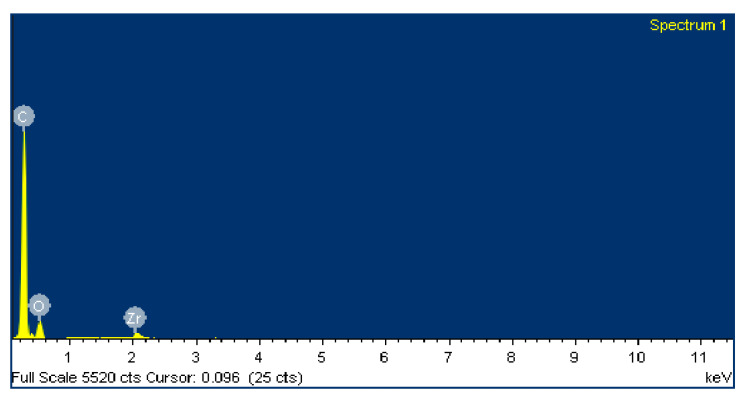
EDS spectrum for zone 2 in the PA46Z sample.

**Figure 18:**
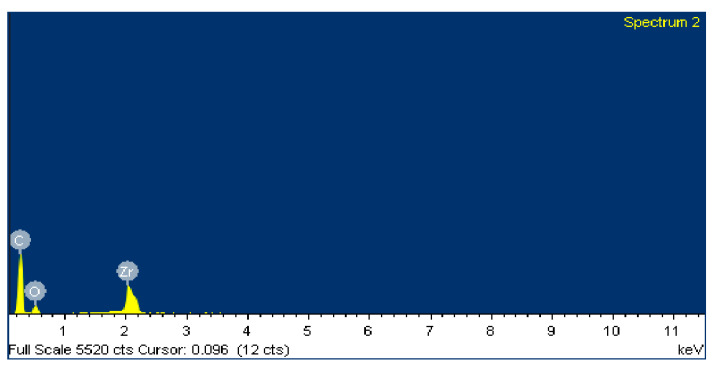
EDS spectrum for zone 2 in a local area marked with an arrow in Figure 16, where ZrO_2_ is located.

Figure 13 shows an SEM image of zone 1, where two EDS analyses were carried out. Figure 14 shows the EDS spectrum for the general area shown in Figure 13, and Figure 15 shows the EDS spectrum for the area marked with an arrow in Figure 13, where white spots of ZrO_2_ appear. Figure 16 shows an SEM image of zone 2, where two EDS analyses were carried out as well. Figure 17 shows the EDS spectrum for the general area shown in Figure 16 and Figure 18 shows the EDS spectrum for the area marked with an arrow in Figure 16, where white spots of ZrO_2_ appear.

In both zones 1 and 2 of the pin sample, white spots appear, indicating the presence of ZrO_2_ particles dispersed into the polymer matrix (Figure 13 and Figure 16). The presence of these particles in zone 1 is confirmed by the identification of Zr and O peaks of the spectra in Figure 14 and Figure 15. Since the spectrum in Figure 15 is obtained directly from a particle of ZrO_2_, the Zr peak intensity is higher than in Figure 14, where the spectrum is calculated for the whole area shown in Figure 13. Figure 17 and Figure 18 also confirm the presence of these ZrO_2_ particles in zone 2, with the appearance of Zr and O peaks in the spectra. These peaks are lower due to the poorer contact between the pin and counterface in zone 2.

EDS spectra in Figure 14 and Figure 15 for zone 1 also show Fe peaks, indicating that the metallic wear particles of the 100Cr6 disc were transferred to the polymeric pin, which supports the hypothesis made from Figure 5d that metallic transfer to the pin had occurred.

**Figure 19:**
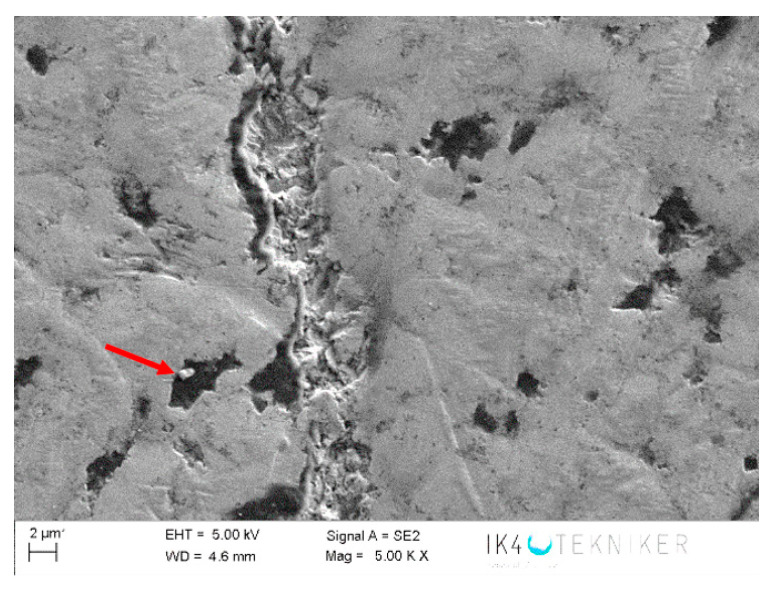
SEM image of a tested area of the disc against a PA46Z pin.

**Figure 20:**
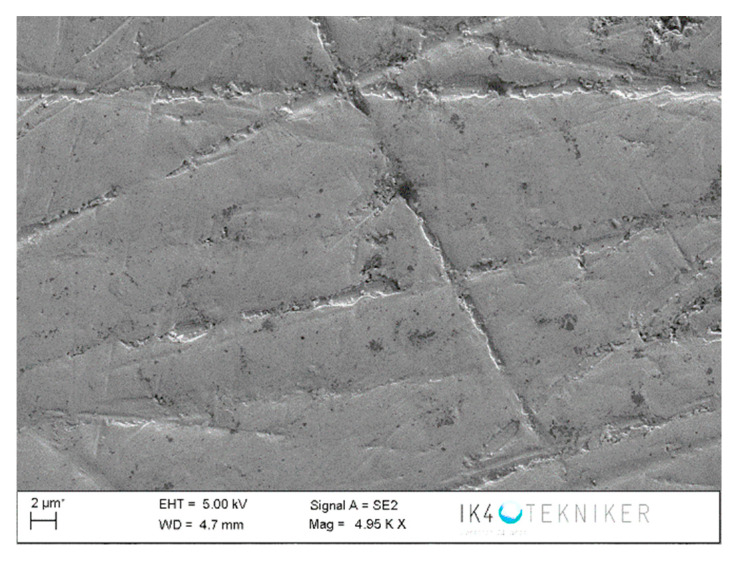
SEM image of a non-tested area of the disc.

**Figure 21:**
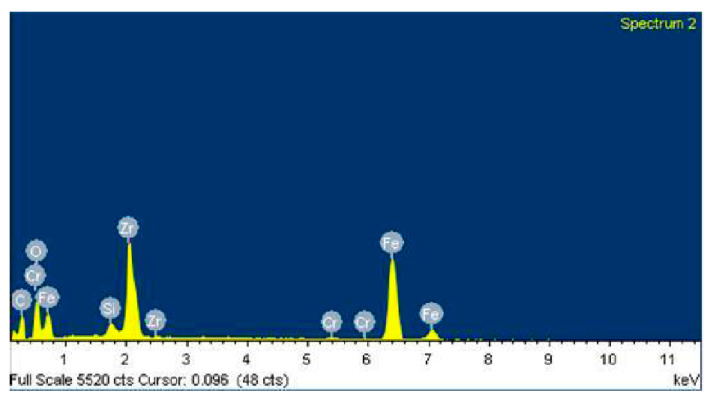
EDS spectrum of the marked area in Figure 19, where traces of ZrO_2_ appear.

The disc counterface area tested against the pin was also analysed by SEM and EDS. It can be observed in Figure 19 that abrasion marks appear, as well as some traces of ZrO_2_ (marked with an arrow). A smoother surface is observed in Figure 20 for areas of the counterface that had not been tested against the pin. To confirm this hypothesis, an EDS analysis was carried out on the area in Figure 19. Figure 21 shows the EDS spectrum for the area marked with an arrow in Figure 19, where traces of ZrO_2_ appear. The spectrum shows peaks of Zr and O, confirming that particles of ZrO_2_ were transferred to the disc. Wear traces in Figure 19 could come from these detached particles of ZrO_2_ transferred to the disc that, due to its high hardness (1200 HV), cause damage to the metallic disc. Figure 20 shows a SEM image of the disc of a non-tested area. It does not show any deep mark of abrasion nor any trace of ZrO_2_.

**Figure 22:**
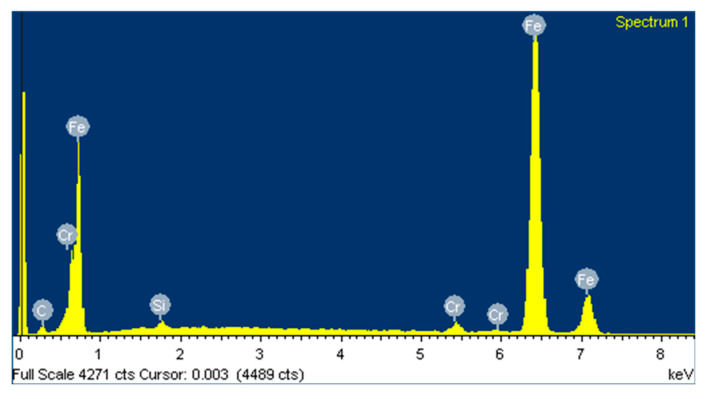
EDS spectrum of a non-tested area of the disc.

**Figure 23:**
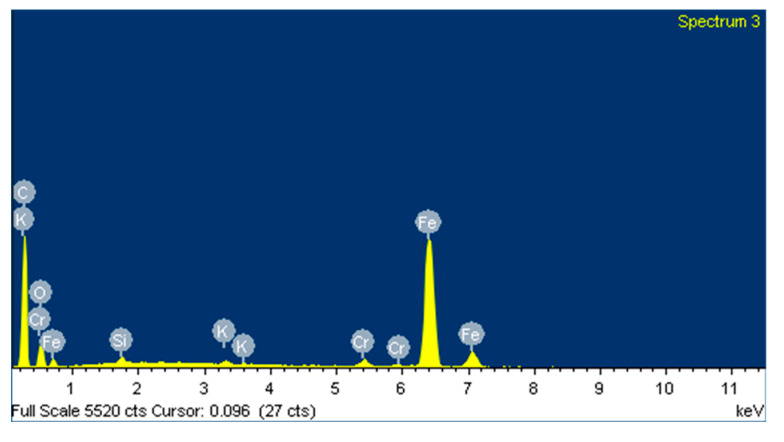
EDS spectrum of a tested area of the disc.

On the other hand, the presence of traces of tribofilm transferred to the disc can be confirmed from EDS analysis on tested and non-tested areas of the disc. Figure 22 shows the composition of a non-tested area of the disc, where low percentages of carbon appear. Figure 23 shows the composition of a tested area of the disc. In this case, the percentage of carbon is higher due to the presence of a polymer tribofilm on the disc surface. This presence of the tribofilm on the disc confirms the adhesive wear mechanism for this kind of tribopair.

## 4. Conclusions

A systematic study comparing the tribological behaviour of different composites based on nylon matrices (PA66, PA46 and PA12) with nanoadditives and reinforcing additives (graphene, graphite, ZrO_2_ and MoS_2_) was carried out.

The results show that the polyamide matrix based on PA12 exhibits lower coefficients of friction for most of the additives tested, graphene, ZrO_2_ and MoS_2_, thanks to their better properties related to higher degrees of crystallinity. On the other hand, PA66 composites showed higher wear rate properties in comparison to PA46 and PA12 composites, mainly due to their poorer properties compared to PA46 and PA12.

The wear mechanism can be defined as adhesive. The presence of nanoadditives modifies the wear mechanism thanks to their higher thermal conductivity, larger surface area and prevention of cracking propagation in the polymer matrix. Graphene and MoS_2_ exhibit better results than graphite for most of the matrices. Although composites doped with ZrO_2_ achieve low coefficients of friction during tests, a transference of metallic particles from the disc to different areas of the polymeric pin is observed. ZrO_2_ particles, detached from the pin, are deposited on the disc, causing abrasion damage and allowing metallic particles to be transferred to the polymeric sample, causing some mild abrasive wear.

## Figures and Tables

**Table 1:** Hybrid composites tested.

Composite Name	Base Polymer	Additive (by Weight)	Additive (by Volume)
PA66GN	PA66	5% graphene	4.10% graphene
PA66GT	PA66	5% graphite	4.20% graphite
PA66M	PA66	5% MoS_2_	1.75% MoS_2_
PA66Z	PA66	5% ZrO_2_	3.60% ZrO_2_
PA46GN	PA46	5% graphene	4.10% graphene
PA46GT	PA46	5% graphite	4.20% graphite
PA46M	PA46	5% MoS_2_	1.75% MoS_2_
PA46Z	PA46	5% ZrO_2_	3.60% ZrO_2_
PA12GN	PA12	5% graphene	4.10% graphene
PA12GT	PA12	5% graphite	4.20% graphite
PA12M	PA12	5% MoS_2_	1.75% MoS_2_
PA12Z	PA12	5% ZrO_2_	3.60% ZrO_2_

**Table 2:** Summary of wear test results for hybrid composites.

Material	μ		Δ*m* (mg)	*w*_s_ 10^−3^ (mm^3^/Nm)
	Average	Standard Deviation		
PA66GN	0.52	0.018	0.35	0.010
PA66GT	0.59	0.027	0.25	0.007
PA66M	0.53	0.051	0.24	0.006
PA66Z	0.49	0.011	0.000 *	-
PA46GN	0.51	0.063	0.11	0.003
PA46GT	0.57	0.030	0.02	0.001
PA46M	0.45	0.014	0.25	0.006
PA46Z	0.58	0.019	−0.06 *	-
PA12GN	0.48	0.010	0.07	0.002
PA12GT	0.52	0.011	0.10	0.003
PA12M	0.41	0.008	0.03	0.001
PA12Z	0.50	0.009	−0.09 *	-

* ZrO2 composites show odd weight results that will be further analysed in this manuscript.

**Table 3:** Summary of EDS spectra for PA46 samples.

Element	Weight (%)					
	Non-Tested Disc	PA46	PA46GT	PA46GN	PA46M	PA46Z
C K	4.49%	34.75%	10.56%	7.12%	10.72%	38.79%
Cr K	1.49%	0.73%	3.48%	1.76%	0.41%	0.34%
Fe K	93.24%	57.63%	50.67%	62.18%	43.65%	22.62%
Si K	0.77%	0.53%	2.02%	2.13%	0.38%	0.30%
O K		5.82%	32.88%	26.29%	44.35%	13.68%
Ca K		0.30%	0.38%			
S K					0.49%	0.30%
Al K		0.24%				
Cl K				0.53%		
Na K						0.86%
Cu K						14.62%
Zn K						8.49%
